# “What We Want Is More Access…”: Experiences of Supportive Cancer Care and Strategies for Advancement in a Canadian Provincial Cancer Care Organization

**DOI:** 10.3390/curroncol28030205

**Published:** 2021-06-15

**Authors:** Jonathan Avery, Hannah K. Schulte, Kristin L. Campbell, Alan Bates, Lisa McCune, Amanda Fuchsia Howard

**Affiliations:** 1 School of Nursing, University of British Columbia, Vancouver, BC V6T 2B5, Canada; Jonathan.avery@ubc.ca; 2 Department of Supportive Care, Princess Margaret Cancer Centre, University Health Network, Toronto, ON M5G 2C1, Canada; 3 Rehabilitation Sciences Graduate Program, University of British Columbia, Vancouver, BC V6T 2B5, Canada; hannah.schulte@ubc.ca; 4 Department of Physical Therapy, University of British Columbia, Vancouver, BC V6T 2B5, Canada; kristin.campbell@ubc.ca; 5 Department of Psychiatry, University of British Columbia, Vancouver, BC V6T 2A1, Canada; Alan.Bates@bccancer.bc.ca; 6 BC Cancer, Vancouver, BC V5Z 1G1, Canada; Lisa.McCune@bccancer.bc.ca

**Keywords:** supportive cancer care, cancer survivorship, qualitative research

## Abstract

Objectives: Despite calls for better supportive care, patients and families still commonly bear significant responsibility for managing the physical and mental health and social challenges of being diagnosed with and treated for cancer. As such, there is increased advocacy for integrated supportive care to ease the burden of this responsibility. The purpose of this study was to understand patient and caregiver experiences with supportive care to advance its delivery at a large provincial cancer care organization in Canada. Method: We used a qualitative descriptive approach to analyze focus groups with patients and caregivers from seven sites across the large provincial cancer care organization. Results: Focus group participants (*n* = 69) included cancer patients (*n* = 57) and caregivers (*n* = 12). Participants highlighted positive and negative aspects of their experience and strategies for improvement. These are depicted in three themes: (1) improving patient and provider awareness of services; (2) increasing access; (3) enhancing coordination and integration. Participants’ specific suggestions included centralizing relevant information about services, implementing a coach or navigator to help advocate for access, and delivering care virtually. Conclusions: Participants highlighted barriers to access and made suggestions for improving supportive care that they believed would reduce the burden associated with trying to manage their cancer journey.

## 1. Background

It is widely recognized that cancer treatments (i.e., chemotherapy, radiation, and surgery) can be physically taxing and emotionally overwhelming for patients and their family caregivers [1]. These multimodal treatments can extend over months and even years. Many patients experience anxiety and depression, cognitive problems (e.g., impaired memory, attention, executive function), loss of appetite, weight loss, constipation, diarrhea, fatigue, nausea, pain, and sleep problems. In addition, people who are diagnosed and treated for cancer often face persistent practical and social problems such as financial concerns, reduced employment opportunities, stigma, social isolation, and spiritual crises [1,2].

To address these challenges, there is an increasing demand for integrated and interdisciplinary teams of healthcare providers to provide care that attends to patient and caregiver-supportive care needs [3,4,5]. Supportive care refers to any programs/services that help improve quality of life throughout the patient experience, from the time of diagnosis and onwards. Supportive care can include psychosocial support (counselling, social work, psychiatry, spiritual health), rehabilitation (physical and occupational therapy, exercise, nutritional guidance, and speech-language pathologists), and pain and symptom management/palliative care. The majority of oncology centres in developed countries have developed and deliver supportive care, though there is variation in the types of supportive care, processes of care delivery, and organizational systems of care. In Canada, healthcare is publicly funded through the Canada Health Act and is implemented through federal, provincial, and territorial relations. Cancer care services are provided within all provinces and territories; however, dedicated entities exist in parallel in many provinces and territories to provide services specific to diagnosis, treatment, and support of cancer patients. Comprehensiveness, design, and delivery of cancer control programs vary between provinces and territories [6]. There is a lack of clarity among Canadian provinces and territories and across cancer programs globally about optimal means of allocating resources and integrating supportive care [7,8]. Thus, evidence, particularly reflecting patients’ and caregivers’ perspectives, is essential for healthcare service re-design.

The study purpose was to describe patients’ and caregivers’ perspectives on the current approach for delivery of supportive care in a large Canadian provincial cancer care organization that provides care to the majority of people diagnosed in the province each year, and how the current care model could be advanced to better meet patient and caregiver needs.

## 2. Methods

We used a qualitative descriptive approach [9] to analyze focus group data gathered from seven sites across the large cancer care organization in the province.

### 2.1. Study Participants

Patients and caregivers were recruited to participate in focus groups of between 6 and 8 participants. The following inclusion criteria were used to recruit patient and caregiver participants: adults ≥ 18 years of age; have a diagnosis of any type of cancer or are a family member or primary caregiver of a patient with a cancer diagnosis; able to provide consent to participate in the research study; and able to provide signed/dated informed consent.

Convenience sampling was used to recruit participants. Clinical staff verbally informed patients of the opportunity to participate in the study with an understanding that participation or non-participation would not affect a patient’s access to, or quality of, care they were receiving. Participants were also asked to extend the invitation to a family member or primary caregiver. Advertisements describing the study were posted at different offices across the provincial cancer care organization as an additional form of recruitment. Potential participants were asked to contact the research coordinator who answered questions, confirmed the participants eligibility, and discussed the consent process. Signed informed consent was obtained in person or via mail prior to scheduling for a focus group.

### 2.2. Setting

Canada has a public healthcare system wherein supportive cancer care is partially paid for by each respective provincial healthcare agency. In the western Canadian province where this study took place, supportive cancer care services are provided through a referral from a registered healthcare provider or by self-referral for some disciplines. Supportive care is divided into three different cancer care programs: (1) psychosocial oncology (counselling, social work, psychiatry, spiritual health, vocational rehabilitation counseling, art therapy); (2) dietitians and speech-language pathologists; (3) pain and symptom management/palliative care (palliative care physicians, nurses, pharmacists).

### 2.3. Data Collection

A total of 7 focus groups were conducted in-person between March and May 2018, each lasting approximately 3 h. All but one focus group were facilitated in English with the other conducted in Cantonese. Each focus group had a primary and secondary facilitator responsible for asking probing questions, encouraging and maintaining respectful and constructive discussions, and keeping the session close to a “naturally occurring interaction” [10]. A focus group guide was used to facilitate an organized discussion around the patient and caregiver experience of supportive care (Table 1). The focus group discussions were audio-recorded and transcribed verbatim by a professional transcription company. The Cantonese transcript was professionally translated into English. A self-reported demographic questionnaire was also completed by each participant prior to their focus group participation.

### 2.4. Data Analysis

Each focus group was analyzed by two independent coders (J.A. and H.S.). Each focus group transcript was read, and notes were made describing initial thoughts and overall impressions of the contents of each focus group. Then, each focus group transcript was reviewed line-by-line and/or in segments to identify and highlight codes and preliminary categories. Constant comparative analysis [11] was used to identify codes and categories and to group these into relevant themes, which were then compared and contrasted. Constant comparative analysis is a method that compares codes, categories, and themes across each individual transcript to develop themes that represent each participant experience. After coding each individual focus group transcript, J.A. and H.S. met to discuss the coding to collaboratively develop and refine a coding framework. This framework was then discussed and reviewed by F.H. and K.L.C. to provide further insight. This process continued until nothing new was being learned about the most relevant themes. Qualitative data analysis software NVivo 10^TM^ version 12 was used to help organize codes, categories, and themes.

## 3. Results

Participant demographics and other descriptive variables are summarized in Table 2. Participants were primarily individuals with a prior cancer diagnosis (*n* = 57) and 12 caregivers also participated. The majority of participants were women (62%), and the average participant age was 63. Overall, participants expressed positive and negative aspects of the supportive care they received along with strategies for improvement. These are depicted in three themes: (1) improving patient and provider awareness of supportive care options services, (2) increasing access, (3) enhancing coordination and integration.

### 3.1. Improving Patient and Provider Awareness of Services

The participants emphasized that they felt ill-informed or not informed about what supportive care programs or services were available to them as part of the cancer care system, such as counselling, peer support, return-to-work information, or nutrition resources. Comments from two participants typified this limited awareness, with the first stating “*… I did not realize that there was as much (support) through the Cancer Centre*” (Site 4) and the second stating “*…we did not know anything about any of these services…*” (Site 5).

This limited awareness arose from a number of circumstances. Participants described moments when they thought certain clinicians lacked the detail or knowledge about available supportive care to make appropriate recommendations or referrals, a sentiment implied by a participant from site 2, who said: “*My oncologist or my surgeon should be aware of some of this stuff… to be aware of the places where we can go and get help*.” At other times, participants felt that the clinicians caring for them did not provide information about available supportive care unless participants took the initiative to ask. In these instances, participants perceived that supportive care was not a priority for a number of clinicians they interacted with, even if they were aware of services, as evident in the comments from a participant from site 3:

*nothing was really offered to me… and I found this giant book that had 80 some odd pages about returning to work. Well, nobody had given it to me. My oncologist hadn’t given it to me. My counsellor hadn’t, and (when) I mentioned it, she says, ‘Oh, I was meaning to give that to you’…*

This lack of being informed contributed to the anxiety felt by many participants as they struggled to find support to cope with being diagnosed and treated for cancer. A participant from site 7 explained that the lack of information about emotional support and cancer rehabilitation contributed to their emotional distress, and as such, they refused to leave their medical appointment until they received some information, saying: “*Eventually I said I’m not leaving until someone explains to me whether it is safe for me to travel on a plane… Why I cannot walk, and how will I be able to walk (again)!*”

Based on these types of experiences, the participants believed that improvements in the dissemination of information specific to supportive care programs and services across all clinicians in the provincial cancer care system could function to reduce patient distress arising from not knowing if and/or how to access supportive care. Other recommendations focussed on synthesizing and curating relevant information to design more practical and efficient ways to talk about supportive care programs and resources during appointments. For example, a participant from site 2 suggested that they should receive “*a little card… that on the front tells you, eat eight to ten servings of fruit and vegetables a day. Exercise 30 min. Don’t drink too much alcohol…Ten points or less…*” and then “… *Have a little conversation about it (information on the card)…*” during their appointment. In addition, the participants alluded to the need for information to be delivered in a way that is inviting and engaging, a sentiment expressed by another participant from site 2, who said: “*…it is frustrating to see that people do not use it (the patient library)… it is in a little corner there by the front door… it does not feel welcoming…*”. Thus, making information easily accessible (i.e., a convenient location that draws the attention of patients and caregivers), and providing information to all patients without requiring a patient request, was essential to reduce the anxiety felt by many participants as they struggled to find support to cope with being diagnosed with and treated for cancer.

### 3.2. Increasing Access

Even when there was knowledge about existing supportive care services and programs, the participants described numerous circumstances when they were unable to access these supports. Strategies to address these barriers to access included making attendance accessible for all and encouraging rather than discouraging access to supportive care.

### 3.3. Making Attendance Accessible for All

The participants frequently described instances when they were unable to attend supportive care programs and services as most were offered in-person and they lived too far from the cancer centre and/or lacked the time and resources to access affordable transportation/parking. In addition, participants did not always feel physically and/or emotionally well enough to attend appointments/programming in person. Poor weather and mobility restrictions, fatigue, anxiety, and depression, and lack of transportation/parking were all listed as factors that decreased the likelihood of attending in-person appointments, groups, or programming. It was not a single factor but rather a combination of factors that together felt insurmountable. A participant from site 2 listed several factors including emotional distress, poor weather, and lack of parking, saying: “*I was coming on a particularly bad day emotionally… It was pouring rain. Moreover, you come and there were no (parking) spots. I’m crying… and I’m 20 min late. Like I had to cancel…*”

To make attendance accessible for all, several participants suggested making transportation and parking more accessible and affordable. Alternatively, many participants suggested removing the need for transportation and parking altogether by developing more virtual/online/telehealth supportive care programming that they could access from their own home/community. Some participants were already accessing virtual services and appreciated the convenience of never having to leave their places of residence to access supports. A caregiver participant from site 7 described this benefit when their family member was accessing pain management support over video conference, saying: “*…she (the cancer patient) wasn’t able to get much out there (in their community), there was no option… so they set up a video conference…*” explaining that “… *there could be (more) advocates (to connect with online)…*” to receive more consistent and additional support without having to commute outside of their community.

However, despite the possible benefits of virtual programming, one participant remained hesitant about accessing this type of care. A participant from site 6 expressed concern that virtual programming could limit access to supportive care for many people because “*unfortunately not everybody has access to Internet…And I do not know about you, but I do not feel real good about putting all my health information on the Internet*…”. Therefore, if more virtual/online programming is provided, issues of Internet access and privacy need to be assured to make access work for all.

### 3.4. Encouraging Rather Than Discouraging Access to Supportive Care

In addition to making access to supportive care work for all, participants described moments when they felt discouraged by their clinicians to access certain types of supportive care. For example, a participant from site 1 felt discouraged when their oncologist dismissed the importance of speech and language pathology, saying: “*The Doctors are very dismissive about anything that has nothing to do with oncology and the chemotherapy… like about the value of speech therapy…*”. Participants also described moments when they were told they needed to be sicker to access certain types of supportive care, including psychosocial support, reflective in the experience described by a participant from site 6: “*…I’m being told, you’re not sick enough so if you want such and such (referring to psychiatric help)…*”. Some participants also believed there was a conflict between the supportive care needs/interests of patients and organizational policies around the types of supportive care that could be provided or promoted through the cancer care system. For example, when discussing their issues around nutritional supplementation, a participant from site 2 stated, “*I think a really important point is that the (organization) has policies about everything. Moreover, those policies are so bureaucratic and are not necessarily in the best interests of the patient*”, prompting them to suggest that “*what we want is more access to help… Or at least somewhere to go to get the help we need…*”.

To help navigate these circumstances, participants thought that, overall, cancer care organizations need to be more patient-centred in their approach to supportive care. A participant from site 4 stressed that “*…we have to recognize it (care) is as a partnership*”, reflecting the importance of having open-ended conversations about different types of supportive care so patients do not feel ignored or dismissed when they discuss or seek the types of supportive care that might interest them. Participants also suggested creating a dedicated supportive care position, an “in-between person” (i.e., a cancer coach or navigator) to help them identify relevant supportive care services or programming and facilitate access within and outside the cancer care system when/if they felt discouraged—an idea expressed by a participant from site 1, who said: “*Maybe having a layperson attending with the medical oncologist or being accessible… because there should be a gatekeeper, a quarterback, a coach*” to encourage rather than discourage accessing the types of supportive care that interests each individual patient.

### 3.5. Enhancing Coordination and Integration

Finally, participants described breakdowns in the coordination and continuity of care. Constantly being referred from one supportive care or medical specialist to another caused emotional strain on participants. As one participant from site 6 noted, “*…I talked to the oncologist. The oncologist said, talk to your GP. I talked to my GP. He sent me to physio. I found it (moving from one healthcare provider to another) tedious…*” to describe the burden of being referred from one specialist to another. A participant from site 5 described their confusion and emotional strain stemming from the contradictory advice they received from different specialists to manage their urinary incontinence, as follows, “*I have my urologist telling me one thing, and I have my oncologist telling me something (else) about a drug to help with my bladder control… this is really concerning… and can be very confusing*”.

To address the strain and confusion associated with moving from one healthcare provider or specialist to another, participants emphasized the need to enhance the integration and coordination of supportive care across all specialties. “*There should be a mandatory consultation process where the oncologist, the pharmacist, they get together, they discuss*…” was an idea pitched by a participant from site 5 to develop a consistent care plan for the patient to reduce unwarranted emotional distress and confusion from a fragmented and siloed cancer care system. Other participants alluded to the benefits of having a “one-stop shop”, a central conduit/place where patients could connect and interact with clinicians and specialists from different areas to obtain a more comprehensive understanding of the different approaches or types of supportive care. A participant from site 4 said: “*doctors and nutritionists and exercise—it was very [integrated]*” when describing the types of care received in the community, concluding that “*I’d love to see that type of thing here (at organization)*”. Creating this type of integration and coordination within and across medical and supportive care specialties was seen as a way to reduce unnecessary confusion and emotional distress for patients.

## 4. Discussion

We have described the patient and caregiver experience of accessing supportive cancer care at a large cancer care organization in a Canadian province and their perspectives on strategies for improvement. Participants experienced a lack of coordination and integration of supportive care with their overall cancer care. This contributed to unwarranted emotional distress and a sense of discouragement when participants attempted to advocate for and/or express their interest in accessing certain types supportive care, such as nutritional guidance, physical rehabilitation services and emotional/psychosocial support. In a systematic review to identify patients’ perceived barriers to using psychosocial oncology services, Dilworth et al. (2014) reported that the most common supportive cancer care deterrents that patients and caregivers encountered were lack of information about services; financial, logistical, and emotional strain associated with transport/travel/parking/to treatment centers; and negative patient and clinician attitudes towards supportive care needs [12]. More recent research has shown similar trends [13,14]. From our study findings, we also drew similar conclusions.

Our study participants highlighted how the lack of information about existing supportive care resources was a significant deterrent to access, which diminished their motivation to seek support. In addition, the lack of information was a source of emotional distress because it left participants feeling helpless and unsure of where/how to seek the support they required either through the cancer centre or in the community. This distress was accentuated in circumstances where participants thought they were discouraged by members of their oncology team to seek out specific types of supportive care such as emotional/psychological support, cancer rehabilitation, and dietary/nutritional counselling.

Although participants in our study did not explicitly state that they were influenced/swayed to access certain types of supportive care over others, previous research has demonstrated that clinician attitudes can fundamentally impact a patients’ ability to access care [12]. For example, in their qualitative focus group study with oncology patients and physicians, Neuman et al. (2010) found that physicians’ and nurses’ subjective belief that supportive care is not integral to services influenced patients’ ability to access said care [15]. Healthcare providers’ subjective beliefs of certain types of need and the importance of certain types of supportive care based on their patients’ gender, age, and ethnicity has also been shown to influence access to symptom management and palliative care services [16,17]. This includes access to cancer care among Indigenous peoples in Canada [18]. Systemic inequalities embedded within cancer care systems is not a new phenomenon; instead, it is considered a foundational concept since how the intersectionality of different social categories could influence access to supportive care has yet to be thoroughly explored in a Canadian context [19].

Although none of our participants suggested an explicit bias towards their care, there was an overall sentiment that clinicians’ attitudes towards certain types of supportive care and/or certain organizational policies around what constitutes appropriate supportive care influenced their ability to access information and resources. These findings substantiate the importance of exploring if/how clinicians’ attitudes and/or institutional policies regarding certain types of supportive care or attitudes towards certain social categories may be influencing access to supportive care.

Suggestions provided by participants to improve integration and coordination included centralizing information about supportive care services, developing virtual/online/telehealth supportive care programming to minimize the need to travel/commute to the cancer centre to access in-person programming, having dedicated professional patient navigators/coaches, and a “one-stop-shop” to access supportive care. These strategies have already been implemented/tested in various ways at different cancer care institutions [20,21,22,23,24]. For example, centralizing information about supportive care services has been developed using eHealth (i.e., web-based Internet search engines or smartphone applications) [24,25]. However, these services are country/region-specific, and the efficacy of these services remains unexplored. These tools would need to be adapted to the specific geographical regions to maximize their potential and to provide information about services in a quick and efficient way [26].

To address navigation and coaching, Howell et al. (2008) proposed and evaluated a nurse-led community-based cancer-supportive cancer model [20]. This nurse-led support model focused on direct care inclusive of teaching/coaching to enhance and then mobilize supportive care services. The individualized and holistic approach to care proposed in this model showed promise in reducing unmet supportive care needs and improving continuity of care and overall health-related quality of life of patients. This model should be tested in future trials to ensure that it can be incorporated into different institutional structures including our own. Other models that integrate a navigator/coach have also been implemented, including primary care physician- and oncologist-led models. A shared care approach has also been proposed, where the responsibility for coordinating and integrating medical and supportive care is shared between specialists and primary care physicians [8]. Evidence varies as to which model is most effective [27], but nonetheless, having a dedicated touchpoint for the patient and caregiver may be a critical component of improving the integration and coordination of supportive cancer care services.

This study has some limitations. Although two individuals independently coded the focus group data, those who facilitated the focus groups were not involved in the data analysis. Their perspectives and observations from the focus group could have provided additional context to clarify the interpretation of the data. In addition, participants were recruited using convenience sampling, leaving the possibility of selection bias. It is also unclear whether there was a relationship between those caregivers and patients that participated in these focus groups. We acknowledge that this is a possible limitation. Combining patients and family caregivers together might affect participant responses given that they typically have different experiences and social positioning. For instance, both patients and family members can often be quite reluctant to share their individual experiences with each other openly and could impact the type of data emerging in each focus group. However, the fact that similar themes were noted across each of the seven focus groups from different treatment sites across the western Canadian province speaks to the commonalities in the experience and the transferability of findings.

In summary, our results pinpoint several directions to advance the delivery of supportive care in cancer. Future research could explore the implementation and feasibility of virtual/online supportive care, coaching/navigation, and developing centralized ways to ensure patients and healthcare providers are aware of what supports exist in each community. In addition, an emerging insight from our focus groups highlights possible systemic concerns around how subjective perceptions of what constitutes appropriate supportive care may be inadvertently denying access to supportive care services. Future research should also explore clinician perceptions to ensure that appropriate and timely access to supportive care is provided to each individual patient.

## Figures and Tables

**Table 1:** Focus Group Guide.

Topic Area	Questions/Probes
Managing physical symptoms and side effects related to your cancer and treatment	This refers to the physical feelings that you had or have related to your experience with cancer. It can include things like pain, nausea, dry mouth, or trouble with eating and physical activity. Let’s share as a group. What are some of the physical feelings that you had during your cancer treatment? How did you cope with the physical feelings? What services did you access to help you manage these physical feelings? What are some areas where you hoped to have more support? Does anyone have anything further to add?
Emotional, social, and spiritual support	This theme refers to a lot of different things. For example, the emotional feelings that you had or have related to your experience with cancer, the relationships with your family and friends, and questions that you had about the meaning of life. Let’s share as a group. What are some of the emotional, social and spiritual experiences that you had during your cancer treatment? How did you cope with these experiences and feelings? What services did you access to help you manage these emotional, social and spiritual experiences? What are some areas where you hoped to have more support? Does anyone have anything further to add?
Practical support	This refers to your practical needs such as financial struggles, travel and transportation for appointments, child care or caregiving that you had or have related to your experience with cancer. Let’s share as a group. What are some of the practical challenges that you had during your cancer treatment? How did you cope with these practical challenges? What services did you access to help you manage these practical challenges? What are some areas where you hoped to have more support? Does anyone have anything further to add?

**Table 2:** Characteristics of focus group participants. Percentages are calculated based on the number of participants who responded in each section.

Characteristic	Participant Has Cancer or Had Cancer (*n* = 57)	Participant Supported or Cared for Someone with Cancer (*n* = 11 *)
Women—no. (%)	36 (63.2)	7 (70.0)
Age—mean yrs (SD)	62.3 (10.7)	64.9 (9.7)
Marital Status—no. (%)		
Single	6 (10.5)	0
Married or Partnered	39 (68.4)	9 (90.0)
Separated or Divorced	9 (15.8)	1 (10.0)
Widowed	3 (5.3)	0
Number in Household (Including Participant Responding)—no. (%)		
Live Alone	16 (28.1)	2 (20.0)
2 People	28 (49.1)	7 (70.0)
3 or More People	14 (22.8)	1 (10.0)
Birthplace—no. (%)		
Canada	35 (64.8)	6 (85.7)
Other, have lived in Canada for less than 5 years	0	1 (14.3)
Other, have lived in Canada for 5 to 10 years	1 (1.9)	0
Other, have lived in Canada for more than 10 years	17 (31.5)	0
Prefer not to answer	1 (1.9)	0
Highest Education Level—no. (%)		
Grade school or less	1 (1.9)	0
High school **	8 (14.8)	0
College or technical school/CEGEP **	19 (35.2)	3 (42.9)
University education (undergraduate or graduate) **	25 (46.3)	4 (57.1)
Prefer not to answer	1 (1.9)	0
Rural or Urban Living Status—no. (%)		0
Rural	3 (5.9)	0
Town (less than 10,000 people)	11 (21.6)	4 (57.1)
City (10,000 or more)	37 (72.5)	3 (42.9)
Employment Status—no. (%)		
Full-time or part-time work	4 (6.6)	1 (14.3)
Paid sick leave/disability leave	18 (29.5)	0
Homemaker/stay-at-home parent	3 (4.9)	0
Full-time student	1 (1.6)	0
Retired	29 (47.5)	4 (57.1)
Unemployed	5 (8.2)	2 (28.6)
Prefer not to answer	1 (1.6)	0
Total Household Income—no. (%)		
Less than $25,000	9 (18.4)	2 (28.6)
$25,000 to less than $75,000	10 (20.4)	2 (28.6)
$75,000 or more	16 (32.7)	2 (28.6)
Prefer not to answer	14 (28.6)	1 (14.3)
Cancer Type of Participant or Patient Cared For—no. (%)		
Bladder	1 (1.5)	1 (9.1)
Blood cancer/haematological	5 (7.6)	0
Brain/Central nervous system	0	1 (9.1)
Breast	15 (22.7)	1 (9.1)
Colorectal (colon or rectal)	10 (15.2)	0
Gynaecological (cervical, ovarian, uterine, or fallopian tube)	7 (10.6)	0
Melanoma skin cancer (not basal cell carcinoma or squamous cell carcinoma)	3 (4.6)	1 (9.1)
Prostate	8 (12.1)	2 (18.2)
Sarcoma	0	1 (9.1)
Stomach or esophagus	0	1 (9.1)
Other cancer type	17 (25.8)	3 (27.3)
Treatment Type of Participant or Patient Cared For—no. (%)		
Surgery	39 (29.8)	5 (33.3)
Chemotherapy (intravenous or oral)	40 (30.5)	3 (20.0)
Immunotherapy/biologic therapy	4 (3.1)	1 (6.7)
Hormone therapy	13 (9.9)	1 (6.7)
Radiation therapy	23 (17.6)	3 (20.0)
Bone marrow or stem cell transplant	1 (0.8)	0
Alternative medicine	8 (6.1)	2 (13.3)
No cancer treatment but close monitoring in case treatment is needed	3 (2.3)	0
Time Elapsed Between Primary Cancer Diagnosis and Focus Group—no. (%)		
Less than 1 year	2 (4.1)	N/A
1–2 years	24 (49.0)	N/A
3–5 years	12 (24.5)	N/A
Greater than 5 years	11 (22.4)	N/A
Chronic Conditions (Before Cancer Diagnosis)—no. (%)		
Arthritis, osteoarthritis, or other rheumatic disease	13 (15.7)	N/A
Cardiovascular or heart condition; hypertension or high blood pressure	17 (20.5)	N/A
Chronic kidney disease	2 (2.4)	N/A
Diabetes	2 (2.4)	N/A
Osteoporosis	1 (1.2)	N/A
Respiratory diseases	5 (6.0)	N/A
Mental health issues	12 (14.5)	N/A
No chronic conditions	17 (20.5)	N/A
Other chronic condition(s)	14 (16.9)	N/A

* One additional individual indicated that they were a caregiver of a deceased cancer patient. However, they did not complete the remainder of the questionnaire. ** Including those individuals who completed some high school, college, or university, respectively, as well as those individuals who completed these programs and received diplomas or degrees.

## Data Availability

The data presented in this study are available on request from the corresponding author. The data are not publicly available due to restrictions (privacy and ethical).
